# Orthodontic considerations for managing patients with functional movement disorders: a narrative review and clinical guide

**DOI:** 10.3389/fdmed.2025.1628802

**Published:** 2025-08-07

**Authors:** Thikriat Al-Jewair, Ajola Zylalaj, Arash Poursattar Bejehmir

**Affiliations:** Department of Orthodontics, University at Buffalo, Buffalo, NY, United States

**Keywords:** functional movement disorder, craniofacial region, orthodontics, spasm, tongue movement

## Abstract

**Background:**

Functional Movement Disorder (FMD) is a neurological condition involving involuntary movements without structural brain or nerve damage. It can significantly affect the craniofacial region, disrupting facial and oral motor functions and complicating dental and orthodontic care. This narrative review outlines the clinical presentation of FMD, emphasizing its relevance in orthodontics and offering a clinical management guide.

**Findings:**

A systematic approach is proposed, detailing strategies from the initial screening visit through active treatment, retention, and post-retention stages. Key strategies include using fixed appliances for better control, scheduling shorter visits to reduce symptom aggravation, and incorporating distraction techniques. Collaborative care with neurologists, psychiatrists, psychologists, physical therapists, and dental professionals is vital, addressing both motor and psychological factors.

**Conclusion and relevance:**

Specialized training, improved diagnostic methods, and customized treatment plans are crucial for managing FMD in orthodontics. These efforts are necessary to optimize care and outcomes for affected patients.

## Introduction

Functional Neurological Disorder (FND) is a condition that presents with a variety of clinical manifestations, including weakness, sensory changes, involuntary movements, gait disturbance, dissociative episodes and speech problems (1). It is described as a multi-network disorder involving abnormalities within and across brain circuits which are responsible for sending and receiving signals across the nervous system (2). FND often resembles organic neurological diseases such as stroke, epilepsy, or multiple sclerosis, but unlike those conditions, FND symptoms arise from disruptions in brain network function, rather than anatomical abnormalities (3, 4).

**Figure 1:**
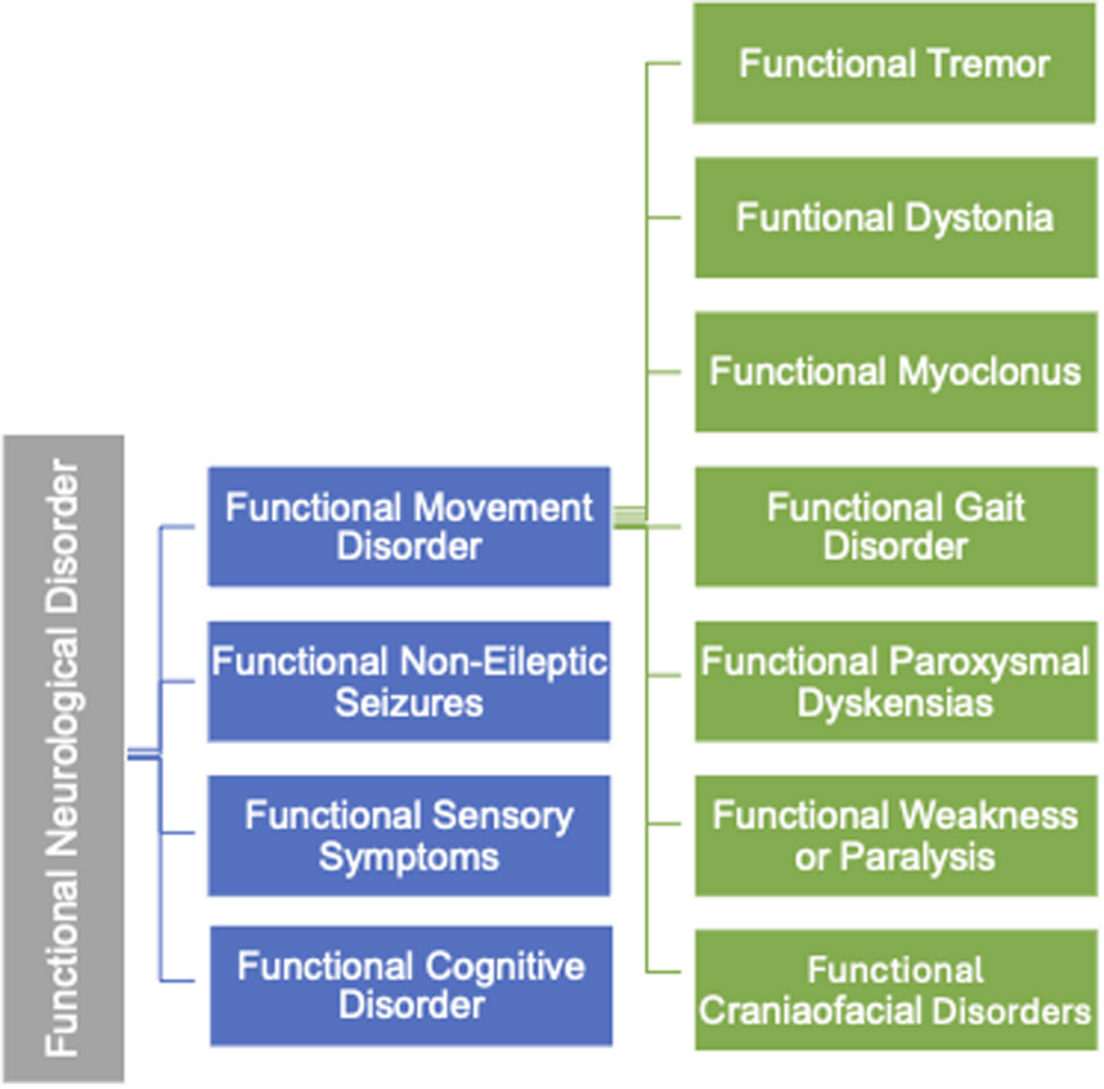
Classifications of FND/FMD.

The variability in clinical manifestations has led to the identification of distinct subtypes within FND, one of which is Functional Movement Disorder (FMD) (5). FMD is a condition where patients experience abnormal, involuntary movements without underlying structural damage to the brain or nervous system. The classification of FMD is illustrated in Figure 1.

## Functional movement disorder

Initially, FMD was classified as a psychogenic disorder and placed under the broader category of conversion disorders. Conversion disorders (CD) are loss or distortion of neurological function that cannot be fully explained by a known organic neurological disease (6). However, modern research has revealed that the FMD disorder is influenced by a combination of neurobiological, psychological, and environmental factors (7). This paradigm shift has led to updates in diagnostic criteria, which no longer require the identification of psychological triggers, though they may play a role in symptom onset (8).

In addition to motor symptoms, the diagnostic criteria for FMD now emphasize sensory processing and the influence of emotional and environmental factors in triggering or intensify symptoms (9). This shift has fostered a more holistic definition of FMD, recognizing its multifactorial nature by integrating both psychological and neurological component. FMD can severely impact patients’ quality of life, often resulting in substantial disability, similar to that observed in organic neurological disorders (10). This narrative review discusses FMD by reviewing its clinical presentation, prevalence, and etiology, management. It also serves as a clinical guide for orthodontists in managing patients affected by FMD within the orthodontic practice.

### Clinical features

The clinical presentation of FMD is highly variable. Common motor symptoms of FMD include tremors, dystonia, myoclonus, gait disturbances jerks, and paresis (11). One of the key characteristics of FMD is its abrupt onset, often triggered by physical or psychological stressors (12). Table 1 summarizes the clinical features of FMD.

**Table 1:** Clinical features of FMD.

FMD subcategories	Clinical features
Functional tremor	• Involuntary shaking movements affecting various body parts. • Variability in amplitude and frequency. (108) • Improvement with distraction or during tasks that divert attention. (109) • Presence of entrainment, where the tremor aligns with the rhythm of voluntary movements. (31)
Functional dystonia	• Sustained or intermittent muscle contractions leading to abnormal postures or repetitive movements. (110) • Fluctuating symptoms that can be inconsistent over time. (111) • Common presentations include inverted ankle postures or clenched fists. (112)
Functional myoclonus	• Sudden, brief, involuntary jerks of muscles or muscle groups. • Variable movements inconsistent with patterns seen in organic myoclonus. (9, 113) • Symptoms may be influenced by psychological factors and can be distractible. (9)
Functional gait disorder	• Abnormal walking patterns, such as dragging a leg, staggering, or exaggerated movements. (114, 115) • Gait abnormalities are often inconsistent and may vary with different situations. (116) • Patients may exhibit ‘knees buckling’ or ‘walking on ice’ patterns. (117)
Functional paroxysmal dyskinesias	• Episodes of sudden, abnormal movements triggered by stress or specific stimuli. (118) • Movements can include dystonia, chorea, or ballistic movements. (119) • Attacks are typically brief and may occur multiple times a day. (120)
Functional weakness or paralysis	• Loss of strength or movement in a limb or limbs without an underlying neurological cause. (121) • A sensation of heaviness affecting one side of the body. (122) • Presence of Hoover's sign; The patient exhibits weakness during voluntary limb movement, but this weakness resolves when the opposite limb moves against resistance. (123)
Functional craniofacial disorders	• Involuntary movements or abnormal postures affecting the head/face regions, and stomatognathic system (70). • May present as hemifacial spasms, convergence spasm, or oromandibular dystonia, etc (124). • Symptoms often include involuntary facial twitching, jaw clenching, or difficulty with speech and swallowing (125). • Movements may be inconsistent, influenced by psychological factors, and can improve with distraction (126).

Functional dystonia is characterized by unusual postures or abnormal muscle contractions, but unlike organic dystonia, these symptoms can change over time or even reverse spontaneously. Symptoms often improve when patients are distracted, but they may worsen when attention is focused on the movement. For example, during a neurological examination, a patient's tremor might decrease or disappear when they are asked to perform a cognitive task, such as counting backwards (13).

Patients with FMD may experience a range of non-motor symptoms, including sensory disturbances, cognitive difficulties such as memory loss, and dissociative experiences (14).

Emotional factors, such as anxiety and depression, are frequently linked to FMD, complicating its clinical presentation. Psychiatric comorbidities can intensify symptoms, leading to a more severe and chronic expression of the disorder (15).

### Prevalence

The true prevalence of FMD remains difficult to estimate due to factors such as underdiagnosis and misdiagnosis. Recent studies suggest that FMD accounts for 2%–20% of patients seen in movement disorder clinics, although this varies based on location and diagnostic criteria applied (16). Epidemiological studies have consistently shown that FMD is more prevalent in women, with a female-to-male ratio of approximately 4:1 (17). The prevalence of FMD phenotypes varies depending on the specific presentation and population. In Western countries, studies report that functional tremor is the most common phenotype, accounting for approximately 36% of cases, followed by functional dystonia at around 34% (18).

FMD typically presents in midlife, between the ages of 35 and 45 years, though this likely underestimates cases in pediatric and elderly populations (19). In pediatric populations, FMD may emerge after acute stressors, such as school-related pressures or family conflicts, while in older adults, it may be misdiagnosed as age-related conditions like Parkinsonism (20). Socioeconomic factors also contribute to the prevalence of FMD, with higher rates observed among individuals from lower socioeconomic backgrounds (21).

### Etiology

The etiology of FMD is complex, involving interactions between several contributing factors, including genetic predispositions, alterations in brain networks, and psychosocial stressors (22).

An emerging area of interest is the role of genetic predisposition. Studies suggest that certain individuals may be more susceptible to FMD due to genetic factors that affect the brain's regulation of motor control and emotional responses (23). For example, variations in genes associated with neurotransmitter systems, particularly those regulating dopamine and serotonin, may increase vulnerability to abnormal movement patterns when exposed to stress or trauma. These findings point to a potential heritable component in FMD, though further research is needed to clarify the specific genetic factors involved (24).

Neurobiological findings also emphasize disruptions in the brain's reward system, particularly involving the dopaminergic pathways. Abnormal dopamine signaling may lead to the reinforcement of abnormal movement patterns, contributing to the persistence of symptoms even after the initial stressor has passed (25).

Social and cultural factors can play a significant role in the development of FMD. Research indicates that societal expectations, cultural norms, and familial influences may shape how symptoms are expressed and perceived (19, 26). In some cultures, certain types of emotional distress may be more likely to manifest as physical symptoms like abnormal movements, reflecting a form of somatization (27). A recent study highlights that prevalence of FMD has increased in recent years, particularly in young individuals, possibly due to socio-environmental factors (28). For instance, the COVID-19 pandemic and increased exposure to social media platforms have been linked to the rise in cases of functional tic-like behaviors, especially in adolescents (29). This suggests that sociocultural and psychological factors may significantly influence the presentation and prevalence of FMD in specific populations. A summary of etiology and risk factors associated with FMD are found on Table 2.

**Table 2:** Etiology and risk factors of FMD.

Category	Etiology/risk factors	Description
Genetic predisposition	Genetic factors affecting brain regulation of motor control and emotional responses.	• Variations in genes regulating dopamine and serotonin may increase vulnerability (24). • Potential heritable component (24).
Neurobiological mechanisms	Disruptions in dopaminergic pathways and reward system regulation.	• Abnormal dopamine signaling reinforces abnormal movement patterns (127). • Links to motor control dysfunction, similar to conditions like Parkinson's disease (functional, not degenerative) (4).
Psychosocial stressors	Psychological stress and trauma exposure.	• Stress may trigger and maintain FMD symptoms (128). • Persistent stress can exacerbate abnormal movements (24).
Socio-cultural influences	Cultural norms, societal expectations, and family influences.	• Certain cultures may be more vulnerable to manifest emotional distress as physical symptoms (19, 26). • Dysfunctional family environments, such as high-conflict households, may increase stress, converted into physical symptoms (129, 130).
Environmental factors	Socio-environmental influences such as the COVID-19 pandemic, and increased exposure to social media.	• Rise in functional tic-like behaviors during the pandemic, especially in adolescents (131). • Influence of social media on symptom expression and perception (131).
Gender	Gender differences in FMD prevalence.	• FMD is more prevalent in women (132). • Hypothesized link to higher susceptibility to psychosocial stressors and environmental triggers (133).
Age	Age-related susceptibility.	• Typically occurs in midlife, 35–45 years (19). • Pediatric cases linked to acute stressors such as school pressures. (134) • Elderly cases often misattributed to age-related conditions like Parkinsonism (135).
Prior illness or injury/trauma	History of physical illness or trauma.	• Physical injuries, such as minor head trauma, may act as a trigger for FMD symptoms in susceptible individuals (136). • In cases with a history of severe emotional or physical abuse, FMD symptoms can emerge as part of a broader functional neurological spectrum (130).

### Diagnosis

The diagnosis of FMD is based on recognizing positive clinical signs, rather than through a process of exclusion (30). A key diagnostic feature is symptom inconsistency, where abnormal movement patterns vary over time in terms of amplitude, frequency, and distribution (31, 32). Additionally, distractibility is an important indicator; abnormal movements often resolve when the patient's attention is directed elsewhere (33).

Functional neuroimaging, particularly fMRI, has become a key tool in diagnosing FMD. Studies have revealed abnormalities in brain regions associated with motor control, most notably the premotor cortex and thalamus, where FMD patients show reduced volume and connectivity (34).

Beyond the premotor cortex and thalamus, research has identified altered activity in regions such as the supplementary motor area (SMA) and basal ganglia, both of which are critical for voluntary motor control (35). Additionally, hyperactivity in emotional processing centers, including the amygdala and cingulate cortex, has been observed in FMD patients, linking emotional dysregulation to movement dysfunction. The increased functional connectivity between motor regions and the emotion-processing areas suggests that FMD arises from a combined disruption of motor and emotional networks (36).

Electrophysiological testing, including electroencephalogram (EEG) and electromyography (EMG), plays an essential role in diagnosing FMD and distinguishing it from other neurological conditions. These tests assess the electrical activity of muscles and nerves, offering valuable insights into movement abnormalities (37). For example, in cases of psychogenic tremor, EMG can detect co-activation of antagonist muscles—a pattern uncommon in organic movement disorders but characteristic of FMD. This simultaneous contraction of opposing muscles results in inefficient or erratic movement, pointing to a functional, rather than structural, abnormality (38).

### Management

Management for FMD incorporates a multidisciplinary approach, blending physical, psychological, neuromodulation, and pharmacological therapies (39).

Physiotherapy plays a pivotal role in the management of FMD. The core components of effective intervention include gaining a comprehensive understanding of the patient's symptoms, assessing the impact on daily function, evaluating the patient's perception and confidence in the established diagnosis, and collaboratively setting clear goals for physiotherapy (40). Research has shown that targeted physiotherapy can be effective in providing sustained symptom relief. In one study, an intensive short-term rehabilitation program led to a 73.5% improvement rate (41).

Occupational therapy, with its holistic approach that addresses physical, mental, and social determinants of health, is also well-suited to assist patients with FMD in maximizing functional outcomes. As in physiotherapy, occupational therapy interventions should be tailored to meet the specific goals of FMD treatment (42).

Neuromodulation therapy represents an exciting and innovative approach for treating complex and challenging cases of FMD, particularly those for whom evidence-based treatment options remain limited. As a novel therapeutic strategy, neuromodulation has the potential to influence key brain networks, positioning it as a promising candidate for addressing the needs of these patients (43).

Transcranial magnetic stimulation (TMS) has long been studied for its ability to noninvasively assess cortical excitability and connectivity (44). Repetitive TMS (rTMS), in particular, has shown potential to produce lasting neuromodulatory effects through mechanisms like long-term potentiation. A recent study involving 33 FMD patients compared TMS applied over the motor cortex contralateral to symptoms against TMS over spinal roots in a control group (45). The observed symptom improvement suggests that nonspecific factors, including behavioral changes or placebo effects, may contribute to the therapeutic response.

Recently, Intermittent Theta Burst Stimulation (iTBS), a form of transcranial magnetic stimulation, has emerged as a potential treatment for FMD. iTBS is a non-invasive brain stimulation technique that involves delivering short bursts of high-frequency magnetic pulses, used to modulate cortical activity in various neurological disorders, including FMD, resulting in reduction of symptoms (46).

The pharmaceutical approach for managing FMD has evolved to encompass a diverse array of agents that target both the symptomatic and underlying neurophysiological abnormalities. By employing various medications, clinicians can tailor interventions to alleviate pain and modulate muscle activity, while improving functional outcomes (47).

Nonsteroidal Anti-Inflammatory Drugs (NSAIDs) are commonly prescribed for pain related to muscular, temporomandibular joint (TMJ) issues, migraines, and trauma (48, 49). They work by inhibiting prostaglandin synthesis, reducing inflammation and pain, but may also slow tooth movement at high doses by affecting bone resorption (50–52).

Selective Serotonin Reuptake Inhibitors (SSRIs) are used to treat depressive and anxiety-related disorders, including those linked to movement issues (53). However, they may alter muscle tone and reduce osteoblastic activity, potentially compromising dental enamel and orthodontic tooth movement (54–57).

Botulinum Toxin (BoNT-A) is utilized for a range of hyperkinetic movement disorders such as cervical dystonia, hemifacial spasm, and tics (58, 59). It reduces muscle hyperactivity in the head and neck but may impact craniofacial growth and mandibular development, especially in children (60–63).

Lastly, dopaminergic and anticholinergic agents, used in conditions like Parkinson's disease, dystonia, and tardive dyskinesia, help improve muscle coordination and oral hygiene (64–67). However, they can also cause xerostomia, increasing the risk for dental caries and tooth structure loss (68, 69).

## FMD in the craniofacial region

In the craniofacial region, FMD commonly impacts the face, eyes, and stomatognathic system, which encompasses the jaws, tongue, lips, palate, teeth and associated soft tissues (70). The symptoms can vary from involuntary facial movements (hemifacial spasm), abnormal eye movements (convergence spasm), and irregular jaw and tongue movements (oromandibular dystonia) (71). Table 3 presents a summary of the craniofacial characteristics of FMD. Studies suggest that FMD in the craniofacial region may be caused by disruptions in the striatothalamocortical circuits, which are responsible for motor control (72).

**Table 3:** Craniofacial characteristics of FMD.

Category	Description
Hemifacial spasms	• Intermittent, involuntary, and often exaggerated contractions of the muscles on one side of the face. (76) • Typically starts around the eye and may spread to other facial muscles. (76, 137) • Can worsen with stress or fatigue. (76, 138, 139) • Often mistaken for tics or other movement disorders. (139)
Convergence spasm	• Intermittent and involuntary over-activation of the eye muscles responsible for convergence. (77) • Symptoms include double vision, eye strain, or headaches. (140) • Temporarily resolves when fixation is relaxed or eyes are closed. (141) • Can mimic other ocular disorders. (142)
Lip pulling/deviation	• Unilateral or bilateral pulling of the lips, often linked with tonic spasms. (5) • May occur spontaneously or during facial expressions. (143) • Can impact articulation or cause social discomfort. (143)
Tongue movement	• Protrusion or deviation of the tongue. (71) • Movements include twitching, thrusting, or rolling. (144) • Often inconsistent with neuromuscular disorders. (5) • May interfere with speech clarity and swallowing. (145)
Functional dysphagia	• Perceived difficulty swallowing. (146) • May include sensations of food sticking in the throat or pain while swallowing. (147) • Can involve fear of choking or psychological factors. (148)
Oromandibular dystonia	• Sustained, intermittent, or task-specific contractions of the stomatognathic muscles. (60) • Involves muscles responsible for chewing, tongue movement, and pharyngeal functions. (149) • Movement patterns include jaw-opening, jaw-closing, jaw-deviation, or tongue-involvement dystonia. (149, 150) • Affects eating and speech. (150)

Hemifacial spasm involves involuntary contractions, or twitching of the muscles on one side of the face (73). The spasms in FMD may appear intermittently and are often inconsistent in terms of frequency and intensity (74). The condition is often triggered by fatigue, anxiety, stress, and symptoms may persist during sleep (75). It can also be accompanied by pain, and in chronic cases, may lead to the development of ipsilateral facial weakness (76).

Convergence spasm has been reported as the most frequent functional eye movement disorder (71, 77). It involves excessive, involuntary contraction of the muscles responsible for eye convergence, resulting in the eyes turning inward with an inability to focus properly, often leading to blurred or double vision (78). It is often misdiagnosed because it can mimic other conditions originating from organic causes, such as abducens palsy (which occurs when the sixth cranial nerve—abducens nerve, is damaged or is not functioning properly) (77).

Oromandibular dystonia (OMD) is a condition that affects the muscles of the jaw, mouth and tongue, causing abnormal jaw clenching, and involuntary mouth and tongue movements, often leading to difficulties in speaking or swallowing (79). OMD are often suppressed during sleep but may intensify with stress, emotional distress, or fatigue (80). OMD can be caused by chronic exposure to antipsychotic drugs, which is referred to as tardive OMD (81). Dopamine receptor blocking agents are the most common drug group implicated in the causation of this condition (82). Also, oromandibular-facial trauma, dental procedures and parotid gland surgery have been reported to exacerbate OMD (83). In clinical practice, botulinum toxin (BoNT) injection is considered to be the most effective treatment for OMD, supported by various small and large scale studies (84, 85).

## Orthodontic implications

FMD can have profound implications in the dental and orthodontic settings, particularly because of the complex interactions between facial muscles and oral structures, which can interfere with routine dental and orthodontic procedures impacting treatment planning, biomechanics and retention protocols and follow ups significantly (60, 86).

### Recognizing the symptoms

Dental professionals should be well-prepared and adequately trained to recognize and differentiate the symptoms of FMD, allowing them to develop and implement tailored treatment plans for these patients. Overlapping symptoms, such as jaw pain, restricted mouth opening, and chewing difficulties are common in both temporomandibular disorders (TMD) and OMD. However, TMD is typically characterized by localized joint pain, often accompanied by clicking or popping sounds during joint movement. It arises primarily from mechanical or structural disturbances affecting the TMJ and associated musculature, frequently linked to parafunctional habits like bruxism (87). Conversely, OMD is a functional movement disorder involving dysfunction in motor control pathways, particularly within the basal ganglia (88). It is marked by involuntary, sustained muscle contractions and abnormal movements, with less emphasis on joint dysfunction.

A distinguishing feature of OMD is the presence of a sensory trick, or geste antagoniste, where simple tactile stimuli, such as touching the chin, can momentarily suppress the dystonic movement (89). This phenomenon is absent in TMD and serves as a valuable clinical indicator to differentiate it from OMD. Additionally, while TMD is predominantly pain-driven, OMD patients primarily experience uncontrollable movements, with discomfort emerging as a secondary consequence of muscle fatigue or joint stress. Importantly, these conditions may coexist or influence one another. The repetitive, involuntary jaw movements in OMD can impose abnormal forces on the TMJ, leading to secondary TMD symptoms such as joint pain or disc displacement (90). On the other hand, chronic TMD-related pain and dysfunction may increase muscle tension and, in susceptible individuals, trigger or exacerbate dystonic patterns (91). Unlike TMD, which follows predictable biomechanical patterns of pain and movement restriction, FMD symptoms may fluctuate with psychological stressors and often lack a clear anatomical basis. The hallmark of FMD includes task-specific manifestations that can transiently improve with distraction or simple maneuvers, further distinguishing them from structural TMJ disorders (92, 93).

### Patient management

FMD is often associated with heightened pain sensitivity, making routine orthodontic adjustments more uncomfortable for patients. It can also cause changes in occlusion due to involuntary muscle contractions, spasms, and abnormal tongue movements, leading to dental misalignment such as open bites, cross bites, and shifting teeth. Parafunctional habits like bruxism and tongue thrusting may further cause enamel wear, tooth mobility, and malocclusion.

To effectively accommodate patients with FMD, dental professionals should begin by establishing clear treatment goals and setting realistic expectations. It is essential to explain any potential risks and complexities in a calm, reassuring manner, while also maintaining thorough and accurate documentation.

During treatment, consider using shorter appointment durations to help prevent fatigue and reduce the risk of overstimulation, both of which can exacerbate functional symptoms. Scheduled breaks can further improve patient tolerance and comfort. Additionally, incorporating distraction techniques such as light, reassuring conversation, calming background music, or sensory tools like stress balls may help reduce anxiety and support a more relaxed experience. Additionally, the use of nitrous oxide sedation during appointments can be an effective option when other pain management techniques prove insufficient.

### Orthodontic mechanics

The sustained or repetitive contractions of the jaw muscles make it difficult for patients to maintain a stable jaw posture during dental treatment (5).

Abrupt head movements and spasms commonly associated with FMD may compromise traditional impression-taking, often requiring multiple attempts due to movement-induced errors. To mitigate this challenge, clinicians may consider alternative modalities, such as intra-oral scanning and cone-beam computed tomography, which offers reduced acquisition time and improved accuracy (94, 95).

Involuntary tongue movements, jaw spasms, and other motor disturbances can dislodge wires, accelerate appliance wear, and require frequent adjustments or replacements, thereby prolonging treatment duration and increasing the risk of dental complications. Similarly, extraoral appliances, such as reverse pull headgear, maybe poorly tolerated by FMD patients. Involuntary movements can destabilize these appliances, causing discomfort and placing excessive stress on dental and craniofacial structures, potentially compromising treatment outcomes.

Given these challenges, fixed appliances may be more suitable for FMD patients, as they provide greater stability and are less affected by involuntary movement-related disruptions.

Indirect bonding techniques can provide significant advantages for FMD patients by reducing chair time during bonding procedures. This approach lowers the risk of accidental events, such as self-inflicted oral injuries or broken brackets from involuntary jaw movements.

Orthodontists should frequently reassess occlusal stability and consider adjustments like occlusal splints. Splints may exert therapeutic effects by modulating sensory signals transmitted via the trigeminal nerve to the sensory trigeminal nucleus (96–99). This nucleus spans from the cervical spinal cord to the mesencephalon and interfaces with reticular interneurons across various levels of the central nervous system. The structural and functional integration of this trigeminal complex may explain the observed overlap between sensory tricks and the use of oral appliances in managing OMD (96). However, the precise mechanisms by which the trigeminal nucleus modulates dystonic movements remain poorly understood. While oral appliances show promise as an adjunct to medical treatments, further research is necessary to clarify their role and optimize their design.

Overall, careful appliance selection and regular monitoring are critical to ensure optimal care and minimize side effects.

### Orthodontic tooth movement

Physiologic orthodontic tooth movement relies on forces of appropriate magnitude and vector to induce bone remodeling on both the compression and tension sides of the periodontal ligament (100). Any disruption in this process can impair orthodontic mechanics and result in imprecise tooth movement. In patients with OMD, unbalanced forces from peri-oral muscles and the masseteric apparatus may counteract the vector-specific forces applied by orthodontic appliances.

Furthermore, high-frequency tremors and unpredictable repetitive forces resulting from orofacial tics may introduce uncontrolled micro forces on teeth, potentially accelerating root resorption in susceptible patients, further complicating orthodontic treatment (101, 102).

### Retention and relapse

Retention is important for patients with FMD, as involuntary muscle forces can lead to relapse even after achieving satisfactory occlusion during orthodontic treatment (103).

Fixed lingual retainers are commonly utilized to counteract the undesirable forces associated with muscle hyperactivity, providing continuous stabilization of the dental arches.

Pharmacologic approaches, such as botulinum toxin (BoNT-A) injections, have shown promise in reducing abnormal muscular activity, which can help improve stability after active orthodontic treatment (104).

These interventions may be beneficial during the retention phase, as they can mitigate the impact of involuntary muscle forces. By integrating appropriate retention strategies and considering adjunctive therapies like BoNT-A, orthodontists can enhance treatment stability and minimize the risk of relapse in FMD patients.

### Effects of medications for managing FMD on orthodontic treatment

Medications used to manage FMD can significantly influence orthodontic treatment outcomes by affecting tooth movement, dentoalveolar and craniofacial growth, retention, and relapse. These effects often result from mechanisms such as altered bone remodeling, muscle activity regulation, and oral health changes like xerostomia and dental attrition (105).

Some medications may slow tooth movement by inhibiting bone turnover, while others can compromise retention by increasing the risk of enamel wear or muscle hyperactivity (106). Understanding these interactions is crucial for orthodontists to make adjustments to treatment plans, minimize complications, and ensure long-term stability, especially in patients requiring prolonged medication use. Table 4 summarizes various medication classes used in managing FMD and their effects on OTM, dentoalveolar growth, retention, and relapse.

**Table 4:** Effects of medications for managing FMD on orthodontic tooth movement, mechanics, retention and relapse.

Medication	Indication	Effects
Nonsteroidal anti-inflammatory drugs (NSAIDs)	• Muscular and TMJ pain (48) • Arthritic conditions (151) • Pyrexia gout (152) • Migraines (153) • Pain associated with trauma. (49)	• Reduce OTM by inhibiting prostaglandin synthesis. (50) • High doses can slow tooth movement by reducing bone resorption. (51) • Short-term, low-dose use is generally safe during orthodontic treatment. (52)
Selective serotonin reuptake inhibitors (SSRIs)	• Dysthymia (154) • Panic disorder (155) • Obsessive-compulsive disorder. (156) • Eating disorders, and premenstrual dysphoric disorder. (53)	• Alter muscle tone, causing enamel loss and dental attrition. (54, 55) • May reduce osteoblastic activity, compromising tooth movement. (56, 57)
Botulinum toxin (BoNT-A)	• Movement disorders (e.g., blepharospasm, cervical dystonia). (157) • Laryngeal dystonia. (158) • Limb dystonia. (159) • Hemifacial spasm. (160) • Focal tics. (61) • Tremor and other hyperkinetic disorders. (58) • Chronic pain conditions and migraine. (59)	• Reduces muscle hyperactivity in head and neck movement disorders. (60, 61) • May affect craniofacial growth by reducing muscle volume and functional load. (62) • Prolonged use in children can affect mandibular development. (63)
Dopaminergic and anticholinergic agents	• Parkinsońs disease. (64) • Tardive dyskinesia. (65) • Dystonia. (66)	• Improve muscle coordination and oral hygiene and overall orthodontic efficiency. (67) • Xerostomia increases the risk of caries and tooth structure loss. (68, 69)

## Challenges and future directions

There are many challenges that complicate the recognition of FMD. Most importantly, it's symptoms often overlap with, and may co-exist alongside, known organic conditions such as Parkinson's disease, multiple sclerosis, and organic dystonias, making diagnosis challenging.

The low prevalence of most FMD cases, combined with limited evidence largely derived from case reports and case series, makes it difficult for oral healthcare professionals to recognize and effectively address FMD manifestations. The absence of standardized treatment guidelines and orthodontic-focused research further complicate these challenges.

Moreover, the lack of structural abnormalities complicates FMD diagnosis, and is requiring clinicians to rely heavily on detailed clinical observation and a thorough patient history. Insufficient training in recognizing FMD among oral healthcare professionals often leads to delayed diagnoses and ineffective treatments, which may worsen the patient's condition.

Addressing these challenges requires a comprehensive approach involving advanced diagnostic techniques, innovative appliance designs, interdisciplinary collaboration, and enhanced training for orthodontists, to improve outcomes and patient experiences. Additionally, advancing research into the pathophysiology of FMD is crucial for improving both diagnosis and treatment. Functional neuroimaging (fMRI) techniques have already demonstrated potential in identifying abnormalities within brain networks responsible for motor control, and advanced investigations should prioritize refining these methods (107).

## Conclusion

Further research on FMD is essential to provide medical professionals with clearer evidence for improved diagnosis and treatment. In orthodontic care, FMD poses significant challenges as involuntary muscle movements can disrupt procedures, compromise occlusal stability, and impact treatment outcomes. FMD's complex etiology, involving motor abnormalities and psychological factors, requires a multidisciplinary approach that goes beyond symptom management.

Accurate diagnosis, particularly in the craniofacial region where FMD can mimic organic conditions, is crucial to avoid misdiagnosis and compromised care. FMD-related muscle hyperactivity can lead to prolonged treatment durations and increased relapse risk.

Table 5 provides insights into specific orthodontic considerations for patients with FMD. Effective management strategies include the use of fixed appliances for greater stability, shorter appointments to minimize symptom exacerbation, distraction techniques, medications, and retention measures like occlusal splints and fixed retainers. Expanding professional training and awareness can promote early recognition and reduce diagnostic errors, ultimately enhancing treatment outcomes and improving the quality of care for FMD patients in orthodontic practice.

**Table 5:** Orthodontic clinical considerations for FMD patients.

Clinical issues/scenarios	Orthodontic clinical considerations
Involuntary repetitive movements interfere with stable head position for intraoral scans or impressions	• Use extra chairside support • Schedule shorter appointments • Consider conscious sedations for longer appointments (e.g., bonding and banding) • Consider indirect bonding • Alternative imaging (e.g., CBCT), if possible
Head and neck jerks disrupt desired orthodontic force vectors and increase relapse or root resorption risk	• Use lighter, more controlled forces, adjust biomechanics • Consider overcorrection • Plan for extended retention and monitoring • Avoid removeable appliances delivering orthodontic forces • More frequent progress follow-ups (e.g., radiographic and clinical)
Medications during treatment may attenuate orthodontic tooth movement efficiency and may cause xerostomia *(e.g., NSAIDs, SSRI, Botox, dopaminergic agents*)	• Collaborate closely with the physician to review medications and modify treatment plans or mechanics accordingly • May consider alternative medications, if possible • Additional preventive oral hygiene measures
*Geste Antagoniste* (sensory trick) can reduce head and neck tremors	• Consider incorporating orthotic appliances to provide sensory input and improve head control during treatment^a^
Communication and informed consent	• Provide clear explanation on risks • Set realistic goals • Document properly

^a^ Needs further investigations and evidence-based recommendations.
